# Zoonotic Transmission of Avian Influenza Virus (H5N1), Egypt, 2006–2009

**DOI:** 10.3201/eid1607.091695

**Published:** 2010-07

**Authors:** Amr Kandeel, Serge Manoncourt, Eman Abd el Kareem, Abdel-Nasser Mohamed Ahmed, Samir El-Refaie, Hala Essmat, Jeffrey Tjaden, Cecilia C. de Mattos, Kenneth C. Earhart, Anthony A. Marfin, Nasr El-Sayed

**Affiliations:** Author affiliations: Ministry of Health, Cairo, Egypt (A. Kandeel, E.A. el Kareem, A.-N. M. Ahmed, S. El-Refaie, H. Essmat, N. El-Sayed);; US Naval Medical Research Unit 3, Cairo (S. Manoncourt, H. Essmat, J. Tjaden, C.C. de Mattos, K.C. Earhart, A.A. Marfin);; US Centers for Disease Control and Prevention, Atlanta, Georgia, USA (A.A. Marfin)

**Keywords:** Influenza, H5N1, zoonotic, zoonoses, respiratory infections, avian influenza, viruses, Egypt, research

## Abstract

During March 2006–March 2009, a total of 6,355 suspected cases of avian influenza (H5N1) were reported to the Ministry of Health in Egypt. Sixty-three (1%) patients had confirmed infections; 24 (38%) died. Risk factors for death included female sex, age >15 years, and receiving the first dose of oseltamivir >2 days after illness onset. All but 2 case-patients reported exposure to domestic poultry probably infected with avian influenza virus (H5N1). No cases of human-to-human transmission were found. Greatest risks for infection and death were reported among women >15 years of age, who accounted for 38% of infections and 83% of deaths. The lower case-fatality rate in Egypt could be caused by a less virulent virus clade. However, the lower mortality rate seems to be caused by the large number of infected children who were identified early, received prompt treatment, and had less severe clinical disease.

## Abstract 2

A lower case-fatality rate may have been caused by a less virulent virus clade.

During January 2003–March 2009, a total of 417 human cases of avian influenza (H5N1) and 256 deaths (61%) were reported worldwide (*1*). Although human-to-human transmission has occurred (*2**–**4*), most human cases have been caused by zoonotic transmission from poultry (*5**–**8*). Investigations have emphasized the need for timely identification to determine demographic groups at risk and activities more likely to cause human infection so that control and prevention measures may be implemented. Such investigations may also determine whether the virus can cause pandemic disease.

Avian influenza (H5N1) in Egypt was first reported in February 2006 when outbreaks were observed in domestic poultry at commercial farms and in backyard flocks in 3 governorates in northern Egypt. Within the first month, avian influenza (H5N1) was detected in 12 other governorates. Despite control measures that included culling, disinfection, vaccination, and controlled poultry movement, epizootic transmission of avian influenza virus (H5N1) continues.

During February 2006–March 2009, avian influenza virus (H5N1) was detected on 907 commercial poultry farms and in 606 backyard flocks. In 2006, poultry farms accounted for 84% of 1,052 outbreaks. In 2007, backyard flocks accounted for 89% of 274 outbreaks. As of March 2009, nineteen of 29 governorates reported infected poultry. The poultry industry in Egypt produces ≈2 million birds per day. Social and economic consequences have been dramatic (losses of ≈2–3 billion US$). Backyard flocks are common; 4–5 million families (≈25 million persons) raise poultry at home. During February 2006–March 2009, a total of 3,941 asymptomatic persons exposed to avian influenza (H5N1) from a person with a confirmed case or from infected poultry were tested by using a real-time PCR; none were positive.

In March 2006, the first human case of avian influenza (H5N1) in Egypt was reported from Qalubiya Governorate (Figure 1). We report the first 63 human cases. We also describe affected demographic groups, illness, mortality rates, and specific events that contributed to transmission.

**Figure 1:**
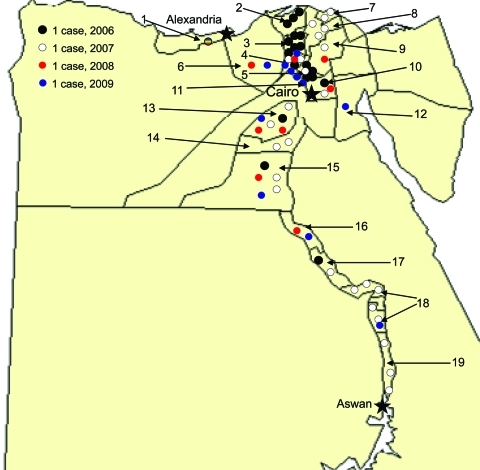
Residences of 63 case-patients with avian influenza virus (H5N1) infections, Egypt, 2006–2009. 1, Alexandria; 2, Kafr El Sheikh; 3, Gharbia; 4, Menofia; 5, Qalubiya; 6, Behera; 7, Damietta; 8, Dakahlia; 9, Sharkia; 10, Cairo; 11, 6th of October; 12, Suez; 13, Fayoum; 14, Benu Suef; 15, Menia; 16, Assyut; 17, Sohag; 18, Qena; 19, Aswan.

## Materials and Methods

The study protocol (NAMRU3.2004.0023) was reviewed and approved by the Naval Medical Research Unit No. 3 Institutional Review Board in compliance with all applicable federal regulations governing protection of human subjects. Suspected cases of avian influenza (H5N1) in humans are reported from all districts in Egypt. A suspected case-patient is a person with influenza-like illness (fever >38°C and 1 of the following signs or symptoms [cough, sore throat, or shortness of breath]) and specific exposure to ill, dying, or dead poultry. All suspected case-patients are referred to specified fever or chest hospitals for testing and medical care. At these hospitals, oropharyngeal swab specimens and serum samples are obtained. Persons with suspected cases receive an initial dose of oseltamivir and are placed in respiratory isolation areas.

Swab specimens are sent to the Ministry of Health (MOH) Central Public Health Laboratory for real-time PCR testing for influenza A virus matrix and H5 genes by using primer–probe sets (*9*). Positive samples are sent to the US Naval Medical Research Unit 3, a World Health Organization H5 Reference Laboratory, for confirmation and virus isolation.

Most case-patients with confirmed avian influenza (H5N1) are transferred to Cairo, Egypt, for care at 2 tertiary hospitals. Data are obtained by healthcare providers who initially evaluate suspected cases and by MOH staff when cases are confirmed. Epidemiologic and clinical data are obtained for each patient with a confirmed case by using a standardized case investigation form. For this study, a medical record review was performed at tertiary care hospitals. Clinical and exposure data were not available for all 63 patients.

The number of patients for whom data were available is noted. Children are defined as persons <15 years of age, adults as persons >15 years of age, delayed hospitalization as >2 days between illness onset and hospitalization, and delayed oseltamivir as >2 days between illness onset and the first oseltamivir dose.

Univariate analyses were performed by using Epi Info version 3.4.1 (Centers for Disease Control and Prevention, Atlanta, GA, USA). Unless otherwise noted, χ^2^ or Fisher exact tests were used. Multivariate analysis to identify risk factors for death was performed by using a backward, stepwise logistic regression model starting with all variables (dichotomized at their median value) significant by univariate analyses. The Wald statistic and log-likelihood ratio were used to exclude variables. Multivariate analyses were performed by using SPSS version 18 (SPSS Inc., Chicago, IL, USA).

## Results

### Overview

**Figure 2:**
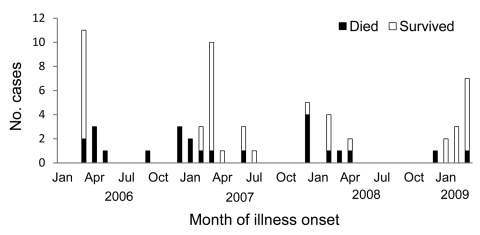
Illness onset for 63 case-patients with confirmed avian influenza (H5N1), by month, Egypt, 2006–2009.

During March 2006–March 2009, a total of 6,355 suspected cases of avian influenza (H5N1) were reported, and samples were tested by the Central Public Health Laboratory. Of these, 63 (1%) cases were confirmed and 24 were fatal (case-fatality rate 38%) (Table 1). Among 63 case-patients, median age was 10 years (range 16 months–75 years), 24 (38%) were women >15 years of age, 5 (8%) were men >15 years of age, 16 (25%) were girls <15 years of age, and 18 (29%) were boys <15 years of age (Table 1). Two infected women were pregnant; both died of respiratory failure. Clinical or exposure data were not available for 2 case-patients (a 31-year-old man infected in Egypt who became ill and whose influenza was diagnosed in Jordan, and a 75-year-old woman who died within hours of hospitalization). Risk factor data on exposure to birds were available for 41 case-patients (Table 1). Confirmed cases were reported from 19 of 29 governorates in Egypt (Figure 1). During 2006, most case-patients were located in the Nile Delta region. In contrast, during 2007–2009, cases were distributed in northern and southern Egypt. Of 63 case-patients, 29 (46%) had illness onset in March (Figure 2).

**Table 1:** Demographic and exposure characteristics for persons with confirmed avian influenza (H5N1), Egypt, 2006–2009

Characteristic	No. (%) persons
Total confirmed cases	63
Deaths	24 (38.0)
Women	40 (63.5)
Age group, y	
0–4	23 (36.5)
5–14	11 (17.5)
15–49	27 (43.0)
>50	2 (3.0)
Exposure (no. persons)*	
Exposure to a confirmed human case before illness (63)	4 (6.3)
Occupational (63)	4 (6.3)
Exposure to likely infected backyard flocks (63)	57 (90.5)
No known exposure (63)	2 (3.2)
Consumption of raw or undercooked poultry products (61)	0
Exposure to likely infected backyard flocks (41)	
Recently purchased domestic poultry from market/seller (41)	12 (29.2)
Recently purchased poultry became ill (12)	7 (58.3)
Noted illness or death among their birds (41)	33 (80.5)
Bred birds (27)	14 (51.8)
Slaughtered birds in past 10 d (27)	13 (48.1)
Defeathered birds in past 10 d (27)	13 (48.1)

*Denominators vary for each exposure because data were not available for all persons.

Three family clusters were identified. The first cluster, reported in March 2006, was composed of 2 siblings (21 months and 6 years of age) in Kafr El Sheikh Governorate. The second cluster, reported in December 2006, was composed of 3 family members (a mother, her daughter, and the daughter’s uncle) in Gharbia Governorate. The third cluster, reported in March 2007, was composed of 2 siblings (4 and 6 years of age) in Qena Governorate. In the first 2 clusters, all shared a common exposure to likely infected poultry and became ill at the same time. In the third cluster, although illness onsets were separated by 4 days, an investigation showed that each child had 2 separate exposures to infected birds. Human-to-human infections were not identified. Household contacts were not given oseltamivir but were followed up closely for 10 days. Secondary infections were not found.

### Clinical Manifestations

Median number of days between illness onset and hospitalization was 2 (range 0–12 days). Female patients and adults were ill longer before hospitalization than were male patients and children. Of 40 female patients, 23 (57.5%) had delayed hospitalization (>2 days after illness onset) compared with 7 (32%) of 22 males. Of 28 patients >15 years of age, 20 (71%) had delayed hospitalization compared with 10 (29%) of 34 patients <15 years of age (p = 0.002). Fever (97%) and cough (72%) were the most common clinical signs. Sore throat was reported by 45% of case-patients and shortness of breath by 25% (Table 2). Adults were more likely to have cough (81%), muscle and joint aches (46%), and shortness of breath (38%); sore throats were more common in children (50%) (Table 2).

**Table 2:** Signs and symptoms at illness onset for 60 persons with confirmed avian influenza (H5N1), by age group, Egypt, 2006–2009*

Sign or symptom	Age group, y, no. (%)		p value
	<15, n = 34	≥15, n = 26	
Fever	34 (100)	24 (92)	>0.05
Cough	22 (65)	21 (81)	>0.05
Shortness of breath	5 (15)	10 (38)	<0.05
Sore throat	17 (50)	10 (38)	>0.05
Vomiting	3 (9)	7 (27)	>0.05
Diarrhea	2 (6)	4 (15)	>0.05
Muscle/joint pain	2 (6)	12 (46)	<0.001
Headache	1 (3)	6 (23)	<0.05
Alteration of consciousness	1 (3)	1 (4)	>0.05

*Data regarding signs and symptoms were available only for 60 of the 63 patients.

### Clinical Course of Disease

Forty-six (73%) of 63 case-patients were transferred to Cairo for definitive care. Of 17 case-patients not transferred to Cairo, 5 (29%) died <2 days after being admitted to a governorate hospital. Of 59 case-patients for whom data on complications were available, >1 secondary complication developed in 25 (42%). Sixteen case-patients had multiple complications (Table 3). The most common complications were acute respiratory distress syndrome (19 cases), shock (14 cases), renal failure (6 cases), and coagulopathy (4 cases). Mortality rate was higher for patients with a complication (n = 25); twenty (80%) died. Only 1 (3%) of 34 patients without complications died (p<0.01).

**Table 3:** Secondary complications in persons infected with avian influenza virus (H5N1), Egypt, 2006–2009*

No. secondary complications	No. persons	Type of complications (no. persons), outcome	Case-fatality rate by no. complications (%)
1	6	ARDS (n = 2), 2 died; bacteremia (n = 2), 2 alive; pneumonia (n = 1), 1 alive; shock (n = 1), 1 alive	2/6 (33)
2	11	ARDS/pneumonia (n = 3), 2 died, 1 alive; ARDS/shock (n = 5), 1 died; ARDS/ renal failure (n = 1), 1 died; ARDS/coagulopathy (n = 1), 1 died; pneumonia/toxic myocarditis (n = 1), 1 alive	9/11 (82)
3	5	ARDS/pneumonia/shock (n = 3), 3 died; ARDS/shock/renal failure (n = 1), 1 died; shock/renal failure/coagulopathy (n = 1), 1 died	5/5 (100)
4	2	ARDS/pneumonia/shock/renal failure (n = 1), 1 died; ARDS/shock/renal failure/coagulopathy. (n = 1), 1 died	2/2 (100)
5	1	ARDS/pneumonia/shock/ renal failure/coagulopathy (n = 1), 1 died	1/1 (100)

*ARDS, acute respiratory distress syndrome.

Of 19 case-patients with acute respiratory distress syndrome, 18 died. The only survivor was an 18-year-old woman who received intubation for 12 days and oseltamivir 2 days after illness onset. A complication was more likely to develop in adults; 20 (77%) of 26 had >1 complication compared with only 5 (15%) of 33 children (p<0.001).

Chest radiographs were reviewed for 58 patients. Twenty-five (43%) radiographs showed lobar infiltrates, of which 20 (80%) were bilateral. Of 33 radiographs for children, 27 (82%) showed no abnormalities. Abnormal laboratory test results included those for leukopenia (16/52, 31%), thrombocytopenia (13/49, 27%), and elevated levels of aspartate aminotransferase (23/46, 50%) and alanine aminotransferase (20/48, 42%).

Medication records were available for 60 patients who received antimicrobial drugs. Of 60 patients, 31 (52%) received oxygen, including 22 (37%) who received mechanical ventilation. Twenty (74%) of 27 adults received mechanical ventilation compared with only 2 (6%) of 33 children (p<0.001). Of 60 patients, 16 (27%) had received corticosteroids. Mortality rates did not differ between intubated patients who received corticosteroids and those who did not.

### Oseltamivir Treatment, Virus Isolates, and Oseltamivir Resistance

All 62 case-patients who became ill in Egypt received >1 dose of oseltamivir. Of 58 patients for whom complete data for oseltamivir was available, 25 (43%) received their first dose <48 hours after illness onset; all but 1 survived. Median duration of treatment was 8 days (range 1–37 days). The first dose of oseltamivir was more likely to be delayed for adults. Twenty (80%) of 25 adults had a delay before receiving oseltamivir compared with 13 (39%) of 33 children (p = 0.005).

Virus isolates were obtained from 34 (54%) of 63 case-patients. Sequencing of hemagglutinin and neuraminidase genes showed all viruses belonged to clade 2.2 and were closely related to isolates from birds in Europe and the Middle East (*10*). Drug sensitivity was determined for all isolates. Resistance to oseltamivir was confirmed in viruses from 2 patients in the same family; both died. Resistance was observed in the initial diagnostic sample and did not occur during treatment. A mutation at position N294S conferring a 12–15× reduction in drug susceptibility was identified in both isolates (*11*).

### Mortality Rates

Of 63 case-patients with confirmed influenza, 24 (38%) died. Median time between onset of illness and death was 9 days (range 4–40 days). Ten (56%) of 18 ill patients died in 2006 compared with 9 (36%) of 25 ill patients in 2007 and 4 (50%) of 8 ill patients in 2008 (Figure 2). Mortality rates were higher for adults and female patients for whom hospitalization or oseltamivir administration were delayed (Table 4). Of 24 deaths, 22 (92%) were among adults and 21 (87.5%) were among female patients. Twenty-one (52.5%) of 40 female patients died compared with 3 (13%) of 23 male patients (p = 0.005). Two (6%) of 34 children died compared with 22 (76%) of 29 adults (p<0.001). Of 30 persons who were ill >48 hours before hospitalization, 20 (67%) died compared with only 4 (12.5%) of 32 persons hospitalized <48 hours after illness onset (p<0.001). Of 33 patients whose first oseltamivir dose was delayed, 19 (58%) died compared with only 1 (4%) of 25 patients who received oseltamivir <48 hours after illness onset (p<0.001).

**Table 4:** CFRs for 63 persons infected with avian influenza virus (H5N1), Egypt, 2006–2009*

Characteristic	Total no.	No. died	CFR, %
Sex†			
F	40	21	52.5
M	23	3	13
Age group, y‡			
0–4	23	0	0
5–14	11	2	18
15–49	27	20	74
>50	2	2	100
All ages	63	24	38
Days between illness onset and hospitalization§			
0–2	32	4	12.5
3–4	12	4	33
5–6	9	8	89
>7	9	8	89
Days between illness onset and first oseltamivir dose¶			
0–2	25	1	4
3–4	14	3	21
5–6	7	5	71
>7	12	11	92

*CFR, case-fatality rate. †p = 0.0004, by χ^2^ test for CFR for female patients vs. that for male patients. ‡p<0.001, by χ^2^ test for patients <15 years of age vs. patients >15 years of age. §p<0.001, by χ^2^ test for patients hospitalized <2 d after illness onset vs. patients hospitalized after 2 d. ¶p<0.001, by χ^2^ test for patients who received oseltamivir <2 d after illness onset vs. patients who received oseltamivir after 2 d.

Although adults were more likely than children to have a delay in hospitalization, age >15 years and delayed hospitalization were independently associated with higher mortality rates. Of 28 adults for whom hospitalization data were available, 12 (43%) were hospitalized in the first 48 hours of illness compared with 27 (79%) of 34 children (p = 0.007). Stratified analysis showed delayed hospitalization was a greater risk factor for death among adults than among children. Eighteen (86%) of 20 adults hospitalized >48 hours after illness onset died compared with 4 (50%) of 8 adults hospitalized <48 hours after illness onset (p = 0.04). None of 24 children hospitalized <48 hours after illness onset died compared with 2 (20%) of 10 children hospitalized >48 hours after illness onset. Nineteen (83%) of 23 female patients hospitalized >48 hours after illness onset died compared with 2 (12%) of 17 hospitalized <48 hours after illness onset (p<0.001).

Eighteen (90%) of 20 adults whose first oseltamivir dose was delayed died compared with 1 (20%) of 5 adults whose first oseltamivir dose was not delayed (p = 0.005). None of 20 children who received oseltamivir <48 hours of illness onset died compared with 1 (8%) of 13 children whose first dose was delayed.

Age, sex, delayed hospitalization, and delayed use of oseltamivir were included in multivariate analysis by using a logistic model to identify risk factors for death. Sex and delayed hospitalization did not contribute to the final model. Because of relatively few cases, high degree of covariance in age, and delayed use of oseltamivir, there was insufficient power to further develop this model. Despite this limitation, analysis showed that age >15 years and having received a first dose of oseltamivir >2 days after illness onset were likely independent risk factors contributing to death.

### Exposure

Handling live domestic poultry likely infected with avian influenza virus (H5N1) was the primary source of exposure. Investigations showed that human-to-human transmission was unlikely; even clusters of case-patients had exposure to infected poultry. Of 63 case-patients, 4 (6%) were involved in poultry production or distribution (3 poultry farm workers and 1 seller), 2 (3%) had unknown poultry exposure, and 57 had direct contact with backyard poultry (Table 1).

Exposure data were available for 41 of 57 case-patients with exposure to backyard flocks. Of these case-patients, 33 (80%) reported having had ill birds in their egg-laying flocks and 12 (29%) had recently bought poultry. Of these 12 case-patients, 7 (58%) reported that purchased birds became ill after being brought home. Of 27 case-patients for whom information was documented, 13 (48%) slaughtered or defeathered birds (Table 1). No case-patients reported eating raw or undercooked animal products.

## Discussion

In February 2006, avian influenza (H5N1) emerged among domestic poultry in the Nile Delta of Egypt. Within 4–5 weeks, it had affected commercial farms and backyard flocks throughout Egypt and resulted in zoonotic transmission to 10 persons in many governorates. Currently, Egypt has reported the third largest number of cases of avian influenza (H5N1) after Indonesia and Vietnam (*1*).

The mortality rate for avian influenza (H5N1) in Egypt (38%) is lower than that in other countries. As of March 2009, mortality rates were 82% in Indonesia, 50% in Vietnam, 66% in the People’s Republic of China, and 68% in Thailand. Explanations for this observation include lower mortality rates for certain demographic groups, clinician awareness resulting in improved medical care, or less pathogenic virus. The most striking finding is the low mortality rate for children. Although children represent 54% of reported infections, they account for only 8% of deaths. This high survival rate is unlikely to be caused by young age alone. Children were hospitalized earlier in the clinical course of their illness, were more likely to receive oseltamivir within the first 2 days, and appeared to be less ill than adults, as noted by the high proportion of chest radiographs with no abnormal findings and the low proportion of children with respiratory failure. Differences in sensitivity of surveillance methods among countries must also be considered.

One must also consider whether the 2.2 virus clade is less virulent. This suggestion is not supported by a report of the 2005–2006 outbreak of clade 2.2 virus (H5N1) in Turkey, where of 8 patients 5–15 years of age, 4 (50%) died (*6*).

Despite overall low mortality rates, particularly among children, the mortality rate in women was >52%. This rate could be due to reasons that include receiving a higher virus inoculum to the lungs through activities associated with slaughtering and defeathering birds, a more profound proinflammatory cytokine response, or delay in receiving healthcare. Only delay in receiving healthcare was examined in this study. Women reported a longer time between illness onset and hospitalization and a longer time until the first dose of oseltamivir than men. Women and men who sought healthcare were admitted to the same facilities and received identical care.

More than 5,000 asymptomatic persons known to have been exposed to poultry infected with avian influenza virus (H5N1) or in contact with confirmed human case-patients were followed up clinically and tested by using real-time PCR. Although prophylaxis was not given, influenza-like illnesses were not observed and all persons showed negative results. Although serologic testing is needed to exclude infection with avian influenza virus (H5N1), it was unlikely that a large proportion of these persons with high-level exposures to infected birds or humans became infected and supports the decision of the MOH to discontinue testing asymptomatic persons. This finding is consistent with those of studies in Thailand (*12*) and Cambodia (*13*).

Although infection and illness do not develop in most persons exposed to infected poultry, all but 2 cases were attributed directly to exposure to poultry likely infected with avian influenza virus (H5N1). No illnesses were attributed to exposure to wild birds. Although 3 family clusters were identified, all 7 persons in these clusters had independent exposures. Many families in Egypt raise backyard flocks for eggs and purchase live poultry for meat. Among case-patients, the likely route of infection appears to be direct handling, slaughtering, or defeathering infected birds recently purchased for meat and mingling of recently purchased birds with egg-laying flocks. Recently purchased birds were frequently slaughtered before illness was noted, and purchase was often followed by illness and death among egg-producing flocks.

Contact between backyard flocks and wild infected birds could not be estimated, but exposure to feral poultry in canals and waterways near affected households was common. Because persons in Egypt rely on live poultry purchased at markets for dietary protein, the price of poultry influences poultry-buying practices of families. Women in several affected families noted exceptionally low prices for healthy looking birds. These prices indicated that they might be buying infected birds. This finding was true when prices of beef increased in response to decreased availability or increased demand. Despite this knowledge, most persons believed they would be able to slaughter and prepare birds before they became ill or died. This belief was true in most cases but recently purchased birds frequently infected egg-laying flocks, which died within days of exposure.

Despite knowledge of overall exposure patterns and identification of groups at risk for exposure, little detailed information on activities that result in infection is available. Although slaughtering and defeathering infected birds appear to be high-risk practices, there have likely been thousands of infected birds sold and slaughtered in homes in Egypt over the past 3 years. Despite this suggestion, we have reports of only 63 cases. Although exposure to avian influenza virus (H5N1) infection is necessary for infection, exposure is not sufficient to explain the epidemiology of cases of avian influenza (H5N1) in Egypt. Whether there is another unknown risk factor or variation in the way women slaughter poultry in Egypt is unclear.

Demographics of influenza cases in Egypt are different from those in other highly affected countries and are useful for determining exposures and activities that result in infection. Women appear to be at greater risk than men of becoming infected, and, once ill, at greater risk of death. In Egypt, the male:female ratio among patients is 1:1.7 and differs markedly from the 1:1 ratio seen globally (*14**,**15*). Caring for or slaughtering poultry is generally the responsibility of women and may explain a higher exposure rate for women. Similarly, age distribution of case-patients differs. In Egypt, 54% of case-patients were <15 years of age, compared with <35% in Indonesia, Vietnam, and China. In Egypt, small children follow their mothers during routine chores, such as feeding and slaughtering poultry. At other times, children will play with poultry, which roam freely around the home. There is a general belief that parents in Egypt will quickly seek medical care for their ill children. This belief is strongly suggested by the fact that children with fever and exposure to dead or ill poultry were consistently evaluated and hospitalized sooner than adults. In addition, many children had mild illness. Mild clinical illness may be caused by early hospitalization, early doses of oseltamivir, or a low virus inoculum.

This report describes 63 human cases of avian influenza (H5N1) in Egypt during March 2006–March 2009. During April–July 2009, a total of 20 additional cases were identified (83 cases by the end of July 2009) for which data were not available. Analysis of limited information reported to the World Health Organization showed a median age of 4 years (compared with 10 years for the 63 cases), a case-fatality rate of 15% (compared with 38%), and faster hospitalization after illness onset. Ongoing transmission in the summer of 2009 is indicative of persistent disease in poultry, and limited analysis reflects the high proportion of influenza in children. Thus, avian influenza virus (H5N1) remains endemic throughout Egypt. However, human infections are rare and disproportionately affect women and their children, who are responsible for caring for and slaughtering birds within the home. To reduce their risk, specific slaughtering practices and other transmission risk factors should be identified and appropriate interventions implemented. In addition, emphasis on controlling domestic poultry populations and increased use of bird cages, hand washing, and other protective measures specific for women and children should continue.
